# The potential role of RNA sequencing in diagnosing unexplained insensitivity to conventional chemotherapy in pediatric patients with B-cell acute lymphoblastic leukemia

**DOI:** 10.1186/s12920-024-01892-w

**Published:** 2024-05-29

**Authors:** Xinyu Li, Zaoli Huang, Liwen Zhu, Weixin Lai, Yunyao Li, Han Chen, Diandian Liu, Junjiu Huang, Dunhua Zhou, Yang Li, Wenjun Weng, Honggui Xu, Luhong Xu, Zhenhua Luo, Jianpei Fang

**Affiliations:** 1 Department of Pediatrics, Sun Yat-sen Memorial Hospital, Sun Yat-sen University, No.107, West Yan Jiang Road, 510120 Guangzhou, Guangdong China; 2 Guangdong Provincial Key Laboratory of Malignant Tumor Epigenetics and Gene Regulation, Sun Yat-sen Memorial Hospital, Sun Yat-sen University, 510120 Guangzhou, China; 3 MOE Key Laboratory of Gene Function and Regulation, State Key Laboratory of Biocontrol, School of Life Sciences, Sun Yat-sen University, 510275 Guangzhou, China; 4 Institute of Precision Medicine, The First Affiliated Hospital, Sun Yat-sen University, 510080 Guangzhou, China

**Keywords:** Acute lymphoblastic leukemia, Children, RNA sequencing, Drug resistance, Gene expression profile

## Abstract

Pediatric B-cell acute lymphoblastic leukemia (B-ALL) is a highly heterogeneous disease. According to large-scale RNA sequencing (RNA-seq) data, B-ALL patients can be divided into more than 10 subgroups. However, many genomic defects associated with resistance mechanisms have not yet been identified. As an individual clinical tool for molecular diagnostic risk classification, RNA-seq and gene expression pattern-based therapy could be potential upcoming strategies. In this study, we retrospectively analyzed the RNA-seq gene expression profiles of 45 children whose molecular diagnostic classifications were inconsistent with the response to chemotherapy. The relationship between the transcriptome and chemotherapy response was analyzed. Fusion gene identification was conducted for the included patients who did not have known high-risk associated fusion genes or gene mutations. The most frequently detected fusion gene pair in the high-risk group was the *DHRSX* duplication, which is a novel finding. Fusions involving *ABL1*, *LMNB2*, *NFATC1*, *PAX5*, and *TTYH3* at onset were more frequently detected in the high-risk group, while fusions involving *LFNG*, *TTYH3*, and *NFATC1* were frequently detected in the relapse group. According to the pathways involved, the underlying drug resistance mechanism is related to DNA methylation, autophagy, and protein metabolism. Overall, the implementation of an RNA-seq diagnostic system will identify activated markers associated with chemotherapy response, and guide future treatment adjustments.

**Supplementary Information:**

The online version contains supplementary material available at 10.1186/s12920-024-01892-w.

## Background

Acute lymphoblastic leukemia (ALL) is the most common malignant disease in children. A high remission rate has been achieved by multiple-drug chemotherapy as an introduction remission therapy for ALL [1]. However, approximately 10% of ALL patients fail to achieve remission, indicating a poor response to chemotherapy and a high risk of relapse and mortality [2]. ALL is also a highly heterogeneous disorder. In a previous large-scale RNA sequencing (RNA-seq) study, according to the transcriptional landscape of B- ALL, B-ALL patients were divided into more than 10 subgroups with different fusion genes and known gene mutations, which can be used to predict patient prognosis and help risk stratification [3]. The underlying genomic defects and mysterious mechanisms of drug resistance are still unclear. Beyond cytogenetics and fluorescence in situ hybridization, gene expression profiling has revealed new cytogenetic subgroups that display specific gene expression patterns. RNA-seq is believed to be implemented within an individual clinical service to enhance the current molecular diagnostic risk classification of leukemia [4–7]. Gene expression pattern-based therapy could be a potential upcoming strategy.

Unfortunately, even though we stratified ALL patients according to genomic information, we did not adjust the therapy greatly. Concerning those without the included fusion gene or known gene mutation, physicians cannot stratify ALL patients without access to the results of the morphology of the bone marrow or minimal residual disease (MRD) at the end of induction therapy [2, 8, 9].

In recent years, small-molecule targeted drugs have been proven effective in clinical trials, regulating a variety of signaling pathways by targeting signaling molecules that play a regulatory role in cell proliferation, differentiation, and apoptosis [10–19]. For those with drug-resistant ALL, further exploration of the gene expression patterns of leukemic cells and translation of gene‒gene interactions would be meaningful for gene expression pattern-based drug combination therapy development. Genome-wide association studies/transcriptome-wide association studies (GWASs/TWAS) of pharmacogenomic/pharmacotranscriptomic markers can identify relevant markers regardless of whether their function was previously known [20], but these studies have low statistical power due to the number of independent tests performed. Fusion gene detection and transcriptome analysis are approaches for identifying candidate pharmacogenetic/pharmacotranscript markers [21].

In this study, by retrospectively analyzing the clinical data on induction chemotherapy in the SCCLG-2016-ALL collaborative group (ChiCTR2000030357) and the relationship between clinical data and gene expression profiles, we evaluated the possibility of developing a novel pattern of personalized combination therapy in a group of children with unexplained chemotherapy-poor ALL patients.

## Methods

### Patient characteristics

Forty-five children with common-B ALL were treated. Bone marrow samples from patients before chemotherapy and when their disease met the criteria for remission according to the SCCLG-2016-ALL protocol were used as test samples and control samples, respectively. The risk classification of this protocol is provided in the supplementary information (SI 1). All patients underwent molecular diagnostic analyses, including karyotype and FISH analysis for *ETV6::RUNX1*, *BCR::ABL1*, and *KMT2A* rearrangements. Our 45 samples represented a selected subset deliberately biased toward samples without a positive molecular diagnostic classification, which explained the poor response to chemotherapy. The clinical and cytogenetic characteristics of the cohort are shown in Table 1 and are inconsistent with the established features of childhood ALL [3, 4].

**Table 1:** Baseline Characteristics of Study Participants

Characteristics	Total
Gender, n(%)	
Male	22(48.9%)
Female	23(51.1%)
Age(y), median(range)	4.6(1.4–14.9)
Age group(y)	
≥ 1, < 10	37 (82.2%)
≥ 10	8(17.8%)
Initial WBC(×10^9^/L), median(range)	12.42(0.49-197.59)
WBC group, n(%)	
< 10 × 10^9^/L	18(40%)
≥ 10 × 10^9^/L, < 50 × 10^9^/L	18(40%)
≥ 50 × 10^9^/L	9(20%)
Initial Hb(g/L), median(range)	69(38–125)
Hb group	
< 60 g/L	13(28.9%)
≥ 60 g/L, < 90 g/L	23(51.1%)
≥ 90 g/Lg/L	9(20%)
Initial PLT(×10^9^/L), median(range)	57.0(4.0-290.0)
PLT group	
< 100 × 10^9^/L	29(64.4%)
≥ 100, < 300 × 10^9^/L	26(57.8%)
Risk group, n(%)	
Non-HR	28(62.2%)
HR	17(37.8%)
Immunophenotype, n(%)	
Common-B	44(66.4%)
Pre-B	1 (6.5%)
CNSL, n(%)	
Yes	0
No	45(100%)
*BCR::ABL1* Status, n(%)	
Negative	41(97.8%)
Positive	4(2.2%)
*KMT2A*-r Status, n(%)	
Negative	45(100%)
Positive	0
*ETV6::RUNX1* Status, n(%)	
Negative	37(82.2%)
Positive	8(17.8%)
Karyotype, n (%)	
Normal	25(55.6%)
Abnormal	20(44.4%)
*IKZF1* deletion, n (%)	
Yes	8(17.8%)
No	37(82.2%)
*P16* deletion, n (%)	
Yes	15(33.3%)
No	30(66.7%)
*RAS* pathway gene mutation, n (%)	
Yes	13(28.9%)
No	32(81.1%)
Prednisone Response, n (%)	
PGR	42(93.3%)
PPR	3(6.7%)
Day 15 BM, n (%)	
M1	33(73.3%)
M2/M3	12(26.7%)
Day 33 BM, n (%)	
M1	45 (100%)
M2/M3	0
Day 15 MRD, n (%)	
< 0.1%	12(26.7%)
≥ 0.1%	33 (73.3%)
Day 33 MRD, n (%)	
< 0.01%	34 (75.5%)
≥ 0.01%	11 (24.4%)

Abbreviations: WBC, white blood cell; Hb, hemoglobin; PLT, platelet; CNSL, central nervous system leukemia; BM, bone marrow; MRD, minimal residual disease; HR, high-risk group; PGR, prednisone good response; PPR, prednisone poor response; M1, M2, M3, neoplasm cells no more than 5% as M1, neoplasm cells more than 5% and no more than 25%  as M2, neoplasm cells more than 25% as M3.

The prednisone response was evaluated on Day 8 after induction by peripheral blood leukemic cell counts. MRD was assessed on Day 15 and Day 33 of induction by flow cytometry. The prednisone response test and MRD status are shown in Table 1. The rates of positive MRD on Day 15 and Day 33 were higher than those in all ALL patients managed in our center, reflecting the selection of nonstandard patients. The included cohort comprised 62.2% non-high-risk and 37.8% high-risk B-ALL patients. None of the included datasets were analyzed in previous publications.

Bone marrow/peripheral blood mononuclear cells were extracted, and total RNA was extracted for quality determination. After qualification, cDNA was synthesized by hybridization, and the original data were obtained by RNA sequencing. The original data were converted by statistical software for statistical analysis, and the differential expression profiles of mRNAs between the B-ALL patients and the bone marrow of patients in remission were obtained. Based on the NCBI Ref Seq, UCSC, RNAdb, and other database resources, bioinformatics analysis was conducted on the chip results, such as Gene Ontology and Pathways, and the gene network map of mRNA was established by calculating the Pearson correlation coefficient.

### Definitions

In this study, risk classification was based on the treatment response to corticosteroids according to the prednisone test and MRD level on Day 15 and Day 33. After seven days of prednisone, prednisone poor response (PPR) was defined as ≥ 1 × 10^9^/L blasts in the peripheral blood. High risk (HR) (*n* = 17) was defined as poor prednisone response (PPR), ≥ 10% MRD on Day 15, and ≥ 0. 1% MRD on Day 33 or relapsed ALL. The remaining patients were defined as non-HRs (*n* = 28).

In the analysis of RNA-seq transcriptome data, we defined upregulated genes as genes whose expression increased twofold and whose Benjamini‒Hochberg adjusted *p* values were greater than those of the control, while downregulated genes were defined as genes whose expression decreased twofold and whose Benjamini‒Hochberg adjusted *p* values were greater than those of the control.

### RNA-seq

Patient blood and bone marrow samples were obtained from the Cancer Tissue Bank of Sun Yat-sen Memorial Hospital. The details of sample handling, RNA extraction, library preparation, and sequencing parameters are provided in the supplementary information (SI 2).

### Fusion detection

RNA sequence fastq.gz files were aligned to the human genome (hg38) using STAR aligner (version 2.7.7a) in 2-pass mode with a parsed version of the comprehensive GENCODE 38 annotation.36. The parameter details are provided in the supplemental materials. Detection of the fusion genes was performed using Arriba v1.1.0 (https://github.com/suhrig/arriba/) with aligned bam files as the input data with default parameters. The visualization of fusion genes was performed using circlize v0.4.13 (https://github.com/jokergoo/circlize).

### Gene expression analysis

RSEM (v1.3.3) was used to calculate the expression levels (reads count, TPM[transcripts per million], FPKM[fragments per kilobase of transcript per million fragments mapped]) of all the genes in each sample and to generate an expression matrix with the bam files generated in the ***Fusion detection******section*** as the input data.

### Gene expression classifier

Classification based on gene expression profiles (TPM values) for each sample was performed using ConsensusClusterPlus [22]. The 5,000 genes with the largest absolute deviations among all samples were selected. The expression levels were normalized to the median. The parameter details of ConsensusClusterPlus are provided in the supplemental materials.

### Differential expression analysis

Differential expression analysis was performed by DESeq2 v1.34.0 with a read count matrix as the input data. The significantly differentially expressed genes were defined as genes with adjusted *p* values less than 0.05 and twofold or greater differences in expression according to the DESeq2 results. Then, all significantly differentially expressed genes were annotated, and pathway analysis was performed with ClusterProfiler (https://github.com/YuLab-SMU/clusterProfiler) or Metascape (https://Metascape.org/).

### Statistical analyses

Comparisons of categorical variables were ascertained by Pearson’s χ2 test or Fisher’s exact test. Two-sided *p* values are reported. Analyses were performed with R (v4.0.2).

## Results

### Clinical characteristics and cytogenetic features of the cohort

The clinical characteristics and cytogenetic features of the cohort are shown in Table 1. In addition to the above information, information on variations in fusion genes, gene mutations, and karyotypes was also collected. Gene variations, including exon deletions or single nucleotide variations, in *CDKN2A* (*n* = 8), *CDKN2B* (*n* = 7), *PAX5* (*n* = 4), *ETV6* (*n* = 3), *E2A* (*n* = 2), *IGH* (*n* = 2), and *BTG1* (*n* = 1) have been reported. Regarding the Ras signaling pathway, *FLT3* (*n* = 5), *KRAS* (*n* = 12), and *NRAS* (*n* = 5) were commonly detected alone or together in 14 patients. Hyperdiploidy of chromosomes + 4, +10, and/or + 17 was detected in 9 patients. *ZNF384-TCF3* fusion (*n* = 1), *EP300-ZNF384* (*n* = 1), and *ZNF384-SYNRG* (*n* = 1) were positive in 3 patients at the first diagnosis. Since most included patients did not have known high-risk associated fusion genes or gene mutations, fusion gene identification was conducted.

### Fusion genes identified in the cohort

**Fig. 1:**
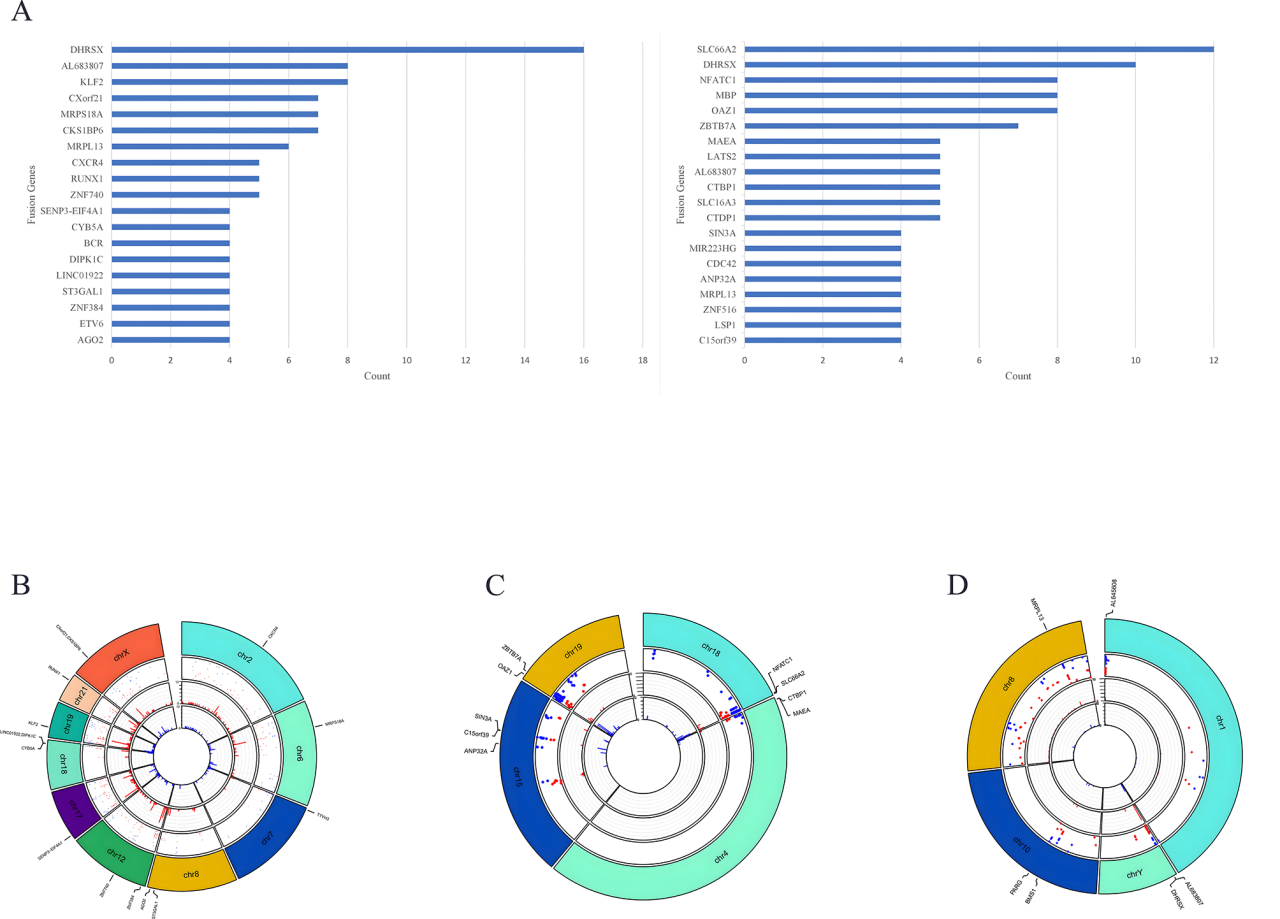
Overview of gene fusions in 45 pediatric B-ALL patients in the cohort. **A**. Illustration of the comparison between fusion break points at onset and at the end of remission induction. **B-D**. The locus distribution of different mutation types on different chromosomes before and after remission

To investigate the gene fusion pattern, we performed RNA-seq analysis of blood and bone marrow samples from this cohort of patients. The most commonly observed breakpoints at gene loci at the disease onset were *FHRSX*, *KLF2*, *AL683807*, *CKS18P6*, *MRPL13*, *ZNF740*, *RUNX1*, and *CXCR4*, while the most commonly observed breakpoints at the end of remission induction were *SLC66A2*, *DHRSX*, *OAZ1*, *MBP*, *NFATC1*, *ZBTB7A*, *CTDP1*, and *SLC16A3* (Fig. 1A). Figure 1B-D shows the distribution of mutations on different chromosomes before and after remission with chromosome coordinates on the horizontal axis. The common mutation locations in the cohort are illustrated in Fig. 1B-D. Gene deletion on chromosome 18 (*CYB5A-LINC01922* and *DIPK1C*), gene duplication on chromosome 8 (*ST3GAl1, AG02*), translocation on chromosome 6 (*MRPS18A*), inversion on chromosome 7 (*TTYH3*), translocation of chromosome 21 (*RUNX1*), translocation of chromosome 12 (*ETV6, ZNF384, ZNF740*), translocation of chromosome 2 (*CXCR4*), translocation of chromosome 19 (*KLF2*), translocation of chromosome X (*CXorf21* and *CKS1BP6*), and translocation of chromosome 17 (*SENP-EIF4A1*) were high-frequency mutations at onset but not during remission (Fig. 1B).

Moreover, mutations of low frequency at onset but high frequency at remission were as follows: duplication of chromosome 18 (*SLGGA2*), inversion on chromosome 4 (*MAEA, CTBP1*), inversion on chromosome 18 (*NFATC1, SLC66A2*), inversion on chromosome 15 (*C1Sorf39, SIN3A, ANP32A*), and inversion on chromosome 19 (*ZBTB7A, OAZ1*). Duplication on chromosome 1 (*AL645608*), duplication on chromosome Y (*AL683807, DHRSX*), inversion on chromosome 10 (*BMS1, PARG*), and translocation on chromosome 18 (*MRPL13*) were not different before and after remission (Fig. 1C-D).

**Fig. 2:**
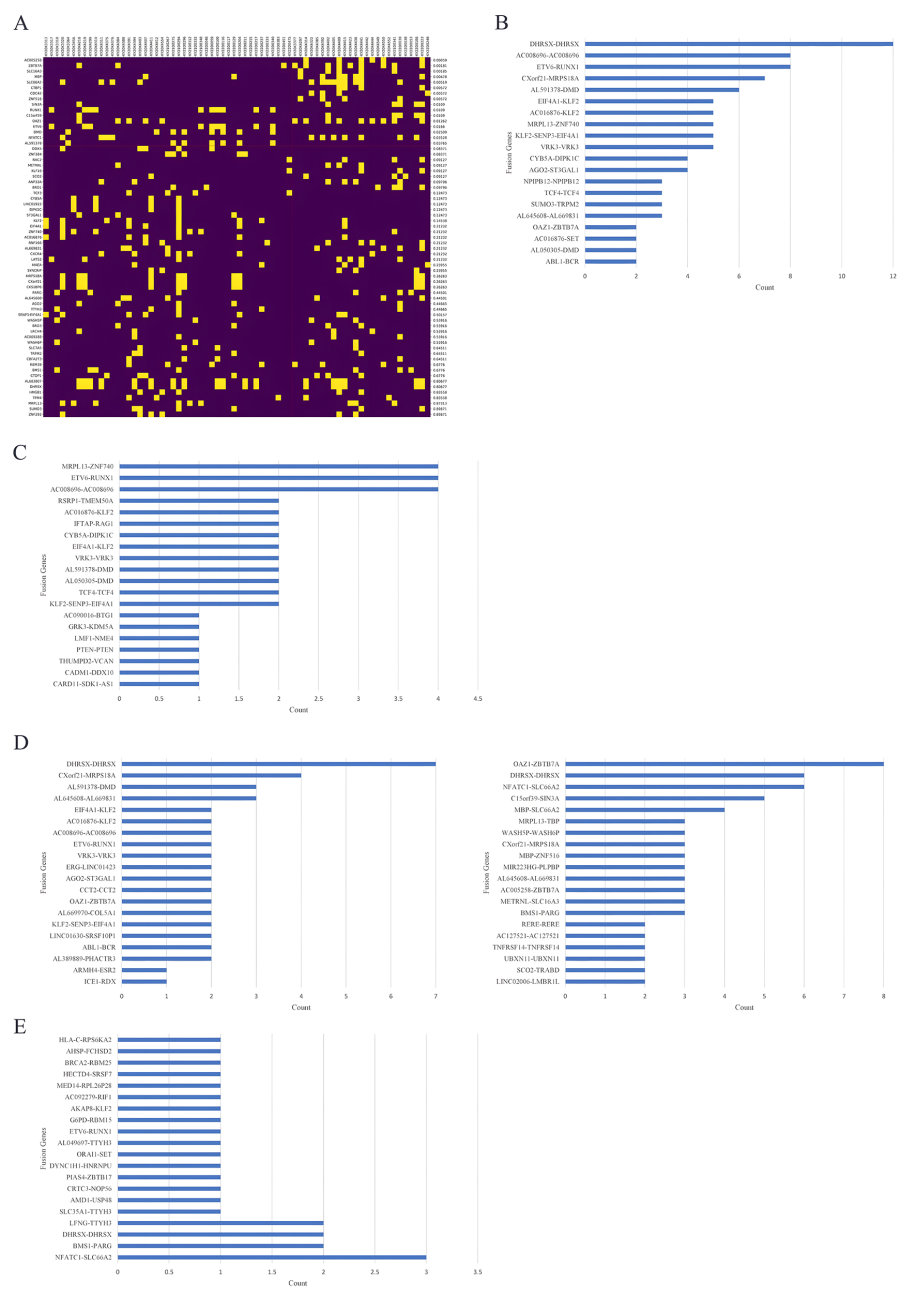
Overview of gene fusions in 45 pediatric B-ALL patients in the cohort. **(A)** Comparison of break points in patients before and after remission. **(B) **Fusion gene pairs in B-ALL detected in the onset samples. **(C)** Fusion gene pairs in non-high-risk B-ALL patients detected in the onset samples. **(D)** Fusion gene pairs in high-risk B-ALL patients detected in the onset samples. **(E)** Fusion gene pairs in B-ALL detected in remission samples

To identify the differences in fusion genes before and after remission, we compared the genes at these breakpoints in patients before and after remission (Fig. 2A). The differences were highly significant for *AC005258* (*p* = 0.00059), *ZBTB7A* (*p* = 0.00181), *SLC16A3* (*p* = 0.00185), *MBP* (*p* = 0.00478), *SLC66A2* (*p* = 0.00519), *CTBP1* (*p* = 0.00572), *CDC42* (*p* = 0.00572), *ZNF516* (*p* = 0.00572), *SIN3A* (*p* = 0.0109), *RUNX1* (*p* = 0.0109), *C15orf39* (*p* = 0.0109), *OAZ1* (*p* = 0.01262), *ETV6* (*p* = 0.0166), *DMD* (*p* = 0.02509), *NFATC1* (*p* = 0.03528), and *AL591378* (*p* = 0.03765).

The fusion gene pair in B-ALL detected in the onset samples is illustrated in Fig. 2B. The most frequently detected fusion gene pairs included *DHRSX-DHRSX* (*n* = 12), *ETV6-RUNX1* (*n* = 8), *C008696-AC008696* (*n* = 8), *CXorf21-MROS18A* (*n* = 7), *AL591378-DMD* (*n* = 6), *VRK3-VRK3* (*n* = 5), *KLF2-SENP3-EIF4A1* (*n* = 5), *MRPL13-ZNF740* (*n* = 5), *AC016876-KLF2* (*n* = 5), *EIF4A1-KLF2* (*n* = 5), *AGO2-ST3GAL1* (*n* = 4), and *CYB5A-DIPK1C* (*n* = 4). The fusion genes involved in non-high-risk B-ALL at the disease onset included *C008696-AC008696* (*n* = 4), *ETV6-RUNX1* (*n* = 4), *MRPL13-ZNF740* (*n* = 4), and *KLF2-SENP3-EIF4A1* (*n* = 2) (Fig. 2C). Several fusion genes, such as *DHRSX-DHRSX* (*n* = 7), *CXorf21-MROS18A* (*n* = 4), *AL591378-DMD* (*n* = 3), and *KLF2-SENP3-EIF4A1* (*n* = 2), were specific to the high-risk group (Fig. 2D). Some of the above fusion genes can still be detected in remission samples, as can some new fusion genes (Fig. 2E).

### Comparison of fusion genes between the high-risk group and the non-high-risk group

According to the initial chemotherapy response defined by the prednisone test and MRD, but not by fusion genes or gene mutations, we classified the cohort into a high-risk group and a non-high-risk group. To determine the differences in biological characteristics between the high-risk group and the non-high-risk group, the differential expression profiles were compared. Clinical characteristics and cytogenetic feature comparisons between the high-risk group and the non-high-risk group are shown in Table 2.

**Table 2:** Baseline Characteristics of Study Participants in the analysis of fusion genes

Characteristics	Non-high risk (*n* = 28)	High-risk (*n* = 17)	
Gender, n(%)			0.763
Male	13(46.4%)	9(52.9%)	
Female	15(53.6%)	8(47.1%)	
Age(y), median(range)	4.6(1.4–12.9)	6.7(2.2–14.9)	0.374
Age group(y)			0.226
≥ 1, < 10	25 (89.3%)	12 (70.6%)	
≥ 10	3 (10.7%)	5 (29.4%)	
Initial WBC(×10^9^/L), median(range)	10.1(1.92–197.6)	12.4(1.61–154.7)	0.721
WBC group, n(%)			0.371
< 10 × 10^9^/L	13(46.4%)	5(29.4%)	
≥ 10 × 10^9^/L, < 50 × 10^9^/L	11(39.3%)	7(41.2%)	
≥ 50 × 10^9^/L	4(14.3%)	5(29.4%)	
Initial Hb(g/L), median(range)	70.0(43.0-125.0)	63.0(39.0–88.0)	0.236
Hb group			0.536
< 60 g/L	8(28.6%)	5(29.4%)	
≥ 60 g/L, < 90 g/L	13(46.4%)	10(58.8%)	
≥ 90 g/L	7(25.0%)	2(11.8%)	
Initial PLT(×10^9^/L), median(range)	56.0(4.0-290.0)	98.0(10.0-217.0)	0.829
PLT group			0.749
< 100 × 10^9^/L	19(67.9%)	10(58.8%)	
≥ 100, < 300 × 10^9^/L	9(32.1%)	7(41.2%)	
CNSL, n (%)			0.651
Yes	1(3.6%)	0	
No	27(96.4%)	15(100%)	
*BCR::ABL1* Status, n (%)			0.564
Negative	25 (89.3%)	16(94.1%)	
Positive	3 (10.7%)	1 (5.9%)	
*KMT2A*-r Status, n (%)			
Positive	0	0	
*ETV6::RUNX1* Status, n (%)			0.583
Negative	23(82.1%)	14(82.4%)	
Positive	5(17.9%)	3(17.6%)	
Karyotype, n (%)			0.135
Normal	13(46.4%)	12(70.6%)	
Abnormal	15(53.6%)	5(29.4%)	
*IKZF1* deletion, n (%)			1.000
Yes	5(17.9%)	3(17.6%)	
No	23(82.1%)	14(82.4%)	
*P16* deletion, n (%)			0.110
Yes	12(42.9%)	3(17.6%)	
No	16(57.1%)	14(82.4%)	
*RAS* pathway gene mutation, n (%)			1.000
Yes	8(26.8%)	5(29.4%)	
No	20(71.4%)	12(70.6%)	
Prednisone Response, n (%)			0.048
PGR	28(100%)	14(82.4%)	
PPR	0	3(17.6%)	
Day 15 BM, n (%)			0.004
M1	25 (89.3%)	8 (47.1%)	
M2/M3	3 (10.7%)	9 (52.9%)	
Day 33 BM, n (%)			
M1	28 (100%)	17 (100%)	
M2/M3	0	0	
Day 15 MRD, n (%)			0.001
< 0.1%	12 (42.9%)	0	
≥ 0.1%	16 (57.1%)	17 (100%)	
Day 33 MRD, n (%)			0.001
< 0.01%	26 (92.9%)	8 (47.1%)	
≥ 0.01%	2 (7.1%)	9 (52.9%)	

Abbreviations: WBC, white blood cell; Hb, hemoglobin; PLT, platelet; CNSL, central nervous system leukemia; BM, bone marrow; MRD, minimal residual disease;PGR, prednisone good response; PPR, prednisone poor response; M1, M2, M3, neoplasm cells no more than 5% as M1, neoplasm cells more than 5% and no more than 25% as M2, neoplasm cells more than 25% as M3.

**Fig. 3:**
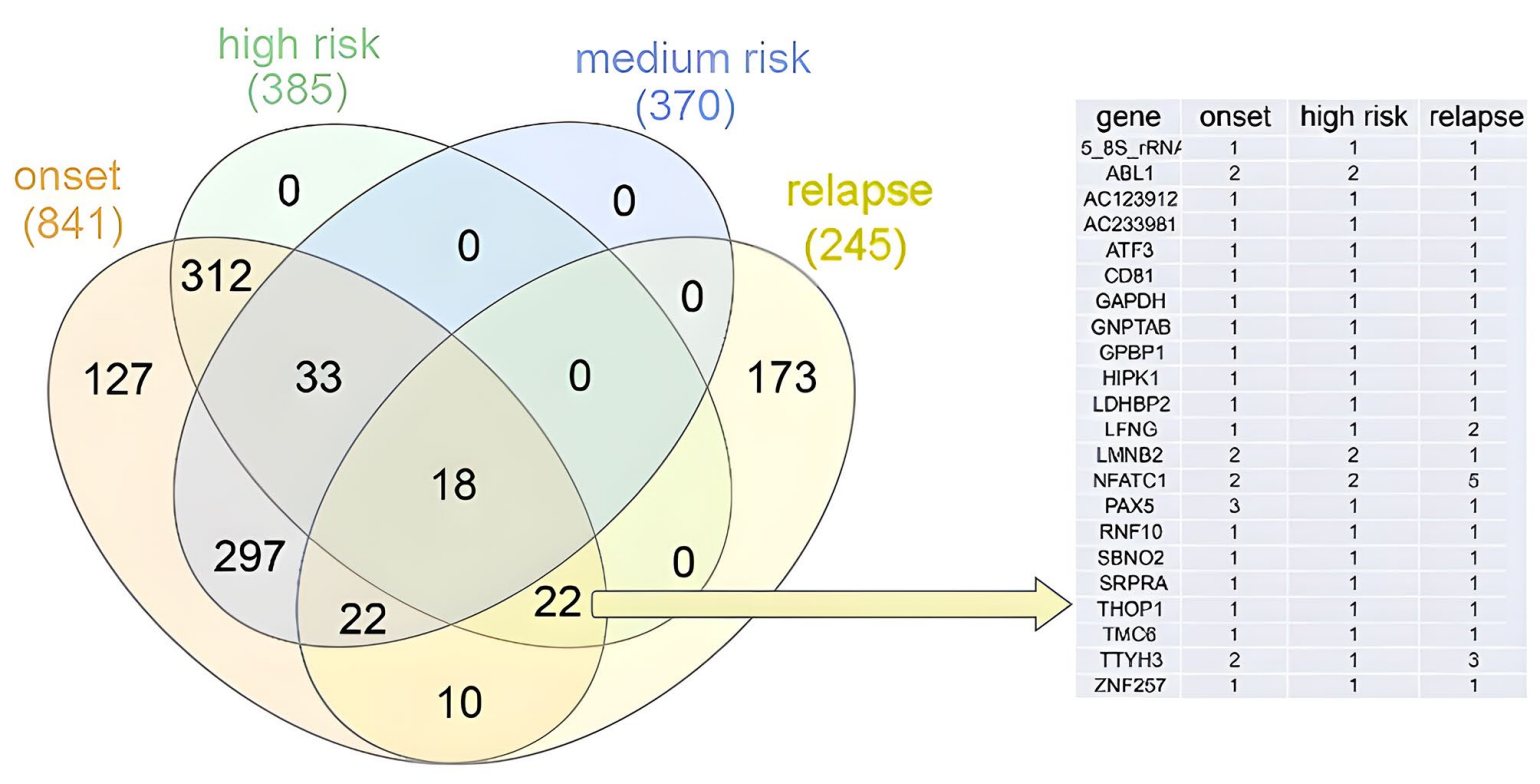
Comparison of differentially expressed fusion genes between the high-risk group and non-high-risk group. The table on the right shows detailed information on 22 genes shared among the high-risk group, onset group and relapse group, but not among the genes in the medium-risk group. The number in the table indicates the number of patients with the corresponding fusion genes

**Fig. 4:**
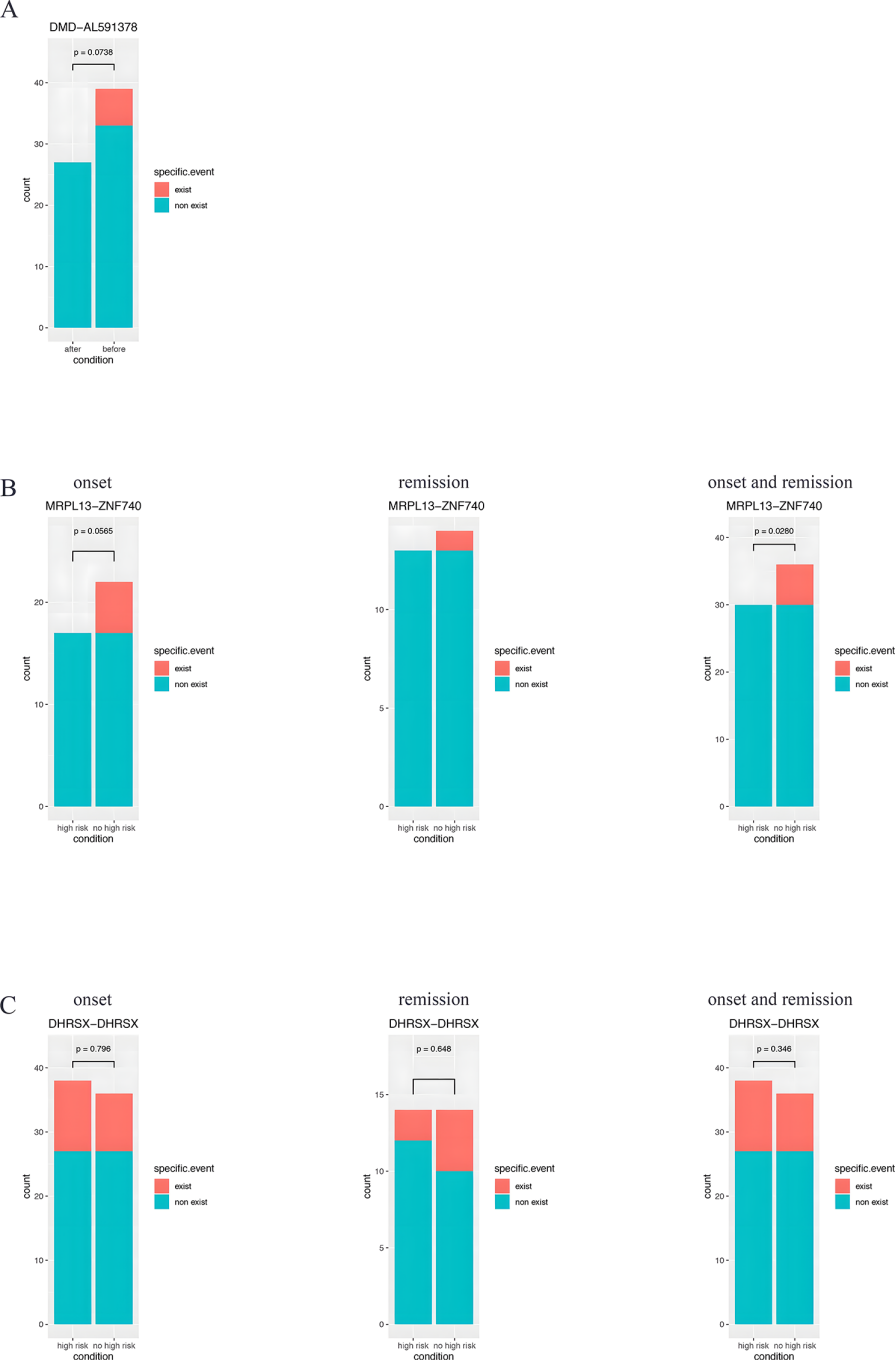
Newly discovered fusion genes with high frequency. **(A)***DMD::AL591378* fusion in onset samples, but not in remission samples. **(B)***MRPL13::ZNF740* fusion in onset samples and remission samples. **(C)***DHRSX::DHRSX* in the high-risk group

To identify potential high-risk-related fusion genes, the genes that were differentially expressed between the high-risk group and the non-high-risk group were further analyzed (Fig. 3). Fusions involving *ABL1, LMNB2, NFATC1, PAX5, and TTYH3* at onset were more frequently detected in the high-risk group, while fusions involving *LFNG, TTYH3*, and *NFATC1* were frequently detected in the relapse group. In addition, there are several newly discovered fusion genes with high frequencys, such as *DMD::AL591378, MRPL13::ZNF740*, and *DHRSX* duplication (Fig. 4). *DMD::AL591378* fusion was frequently found in onset samples and disappeared at remission (Fig. 4A). *The MRPL13::ZNF740* fusion was frequently found in the non-high-risk group at onset, but it did not disappear after treatment (Fig. 4B). *DHRSX-DHRSX* presented a high frequency in the high-risk group (Fig. 4C). *DHRSX-DHRSX* is *AL683807.2* (442,713) and *DHRSX (8017)-DHRSX*. During analysis, when fusion sites are located between genes, upstream or downstream genes are selected according to the location of fusion sites in the transcript to represent the fusions. In the output result, for *DHRSX-DHRSX, AL683807.2*(442,713) is the only form, while *DHRSX (8017)-DHRSX* does not exist. According to the results, at the locus 8017 upstream of *DHRSX*, there is a fusion within the *DHRSX* gene, which is a duplication.

### Elevated gene expression and low gene expression in the cohort

**Fig. 5:**
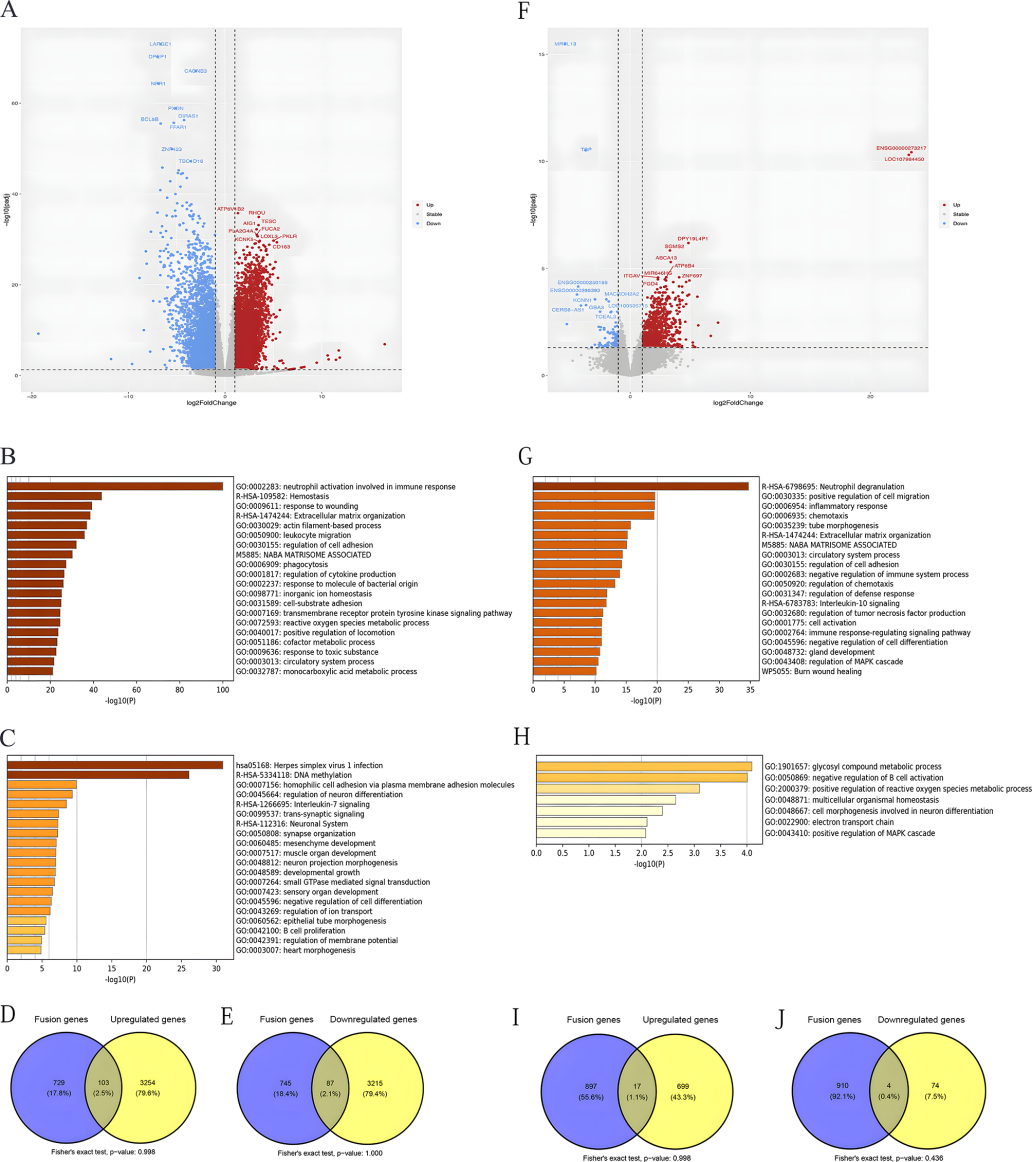
Genetic expression alterations in 45 pediatric B-ALL patients in the cohort. **(A)** Volcano plot comparing the gene expression profiles before and after remission. **(B)** The most highly upregulated pathways. **(C)** The most downregulated pathways. **(D)** Venn diagram showing the overlap between fusion genes and upregulated genes after remission. **(E)** Venn diagram showing the overlap between fusion genes and downregulated genes after remission. **(F)** Volcano plot comparing the gene expression profiles in the high-risk group before and after remission. **(G)** The most highly upregulated pathways in the high-risk group. **(H)** The most downregulated pathways in the high-risk group. **(I)** Venn diagram showing the overlap between fusion and upregulated genes in the high-risk group after remission.** (J)** Venn diagram showing the overlap between the fusion genes and downregulated genes in the high-risk group after remission

Compared to the gene expression profile before remission, 3824 genes were significantly upregulated and 5853 genes were significantly downregulated at remission (Fig. 5A). The differentially expressed genes were annotated functionally. The most highly upregulated pathways are illustrated in Fig. 5B and are mainly involved in the activation of neutrophils in the immune response, hemostasis, and stress response. The most downregulated pathways are illustrated in Fig. 5C and are involved mainly in herpes simplex virus infection and DNA methylation. DNA methylation played a very special role in this cohort. Moreover, the genes related to homophilic cell adhesion via plasma membrane adhesion molecules, development growth, regulation of cell differentiation, and B-cell proliferation were involved in leukemia progression in the cohort. By comparing the overlap between these DEGs and fusion genes, we identified several shared genes (Fig. 5D and E). However, the overlaps were not significant.

### New classifier for identifying subgroups in B-ALL patients

There were 102 upregulated genes and 56 downregulated genes in the high-risk group (Fig. 5F). The pathways involved are illustrated in Fig. 5G-H. Pathways related to exocytosis, cell chemotaxis, inflammatory response, and cell proliferation and survival were ultimately upregulated. These differences are likely attributed to the leukemia stem cells and relapse clones retained in the high-risk group. The pathways related to the regulation of ion transmembrane transport, potassium ion transmembrane transport, negative regulation of cellular amide metabolic processes, and protein‒DNA complex assembly were suppressed, which likely suppressed protein metabolism. By comparing the overlap between these DEGs and fusion genes, we identified several shared genes (Fig. 5I and J). However, the overlaps were not significant.

**Fig. 6:**
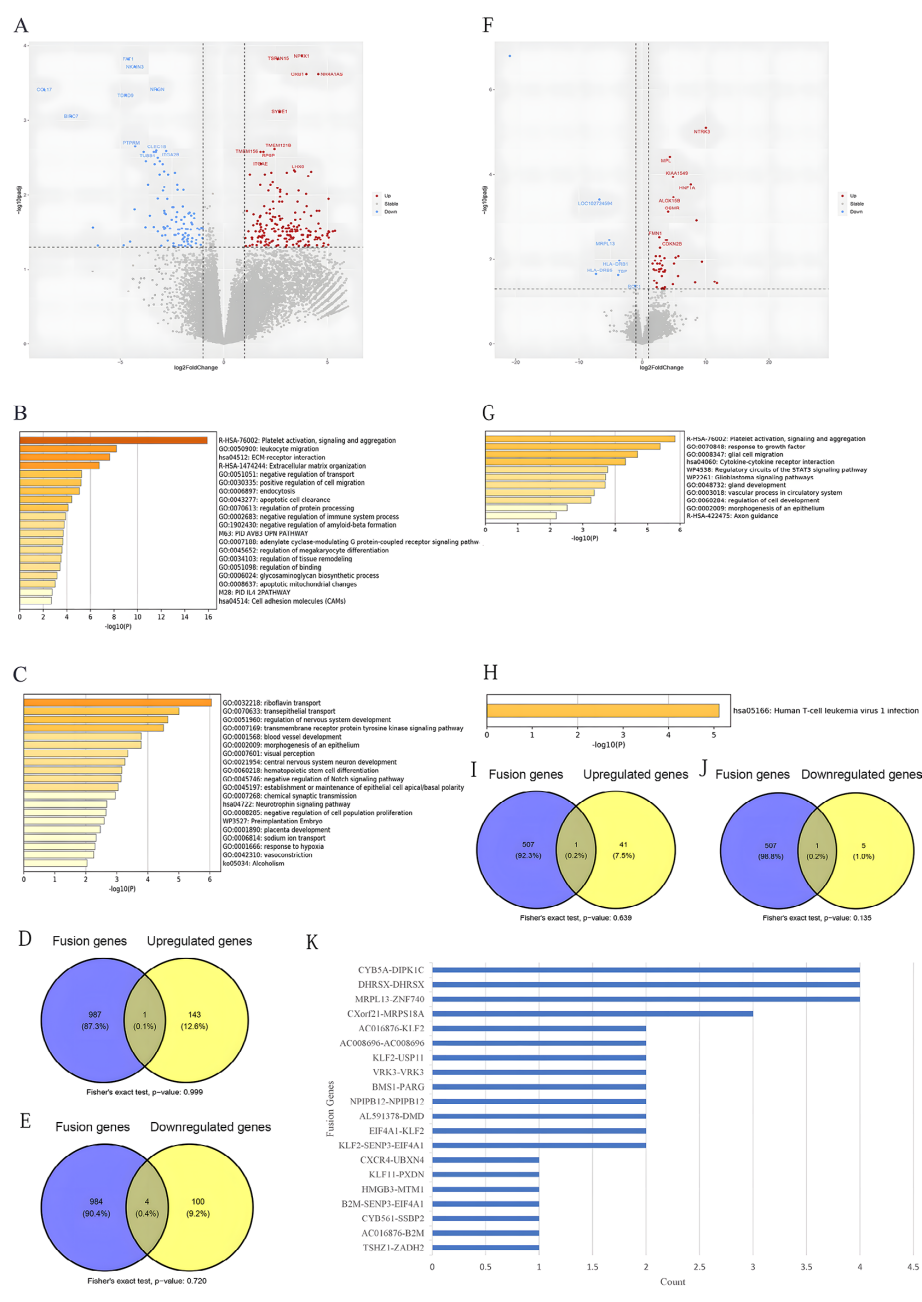
Genetic expression alterations in 13 RAS signaling pathway mutated pediatric B-ALL patients in the cohort. **(A)** Volcano plot comparing the gene expression profiles before and after remission. **(B) **The most highly upregulated pathways. **(C) **The most downregulated pathways. **(D)** Venn diagram showing the overlap between fusion genes and upregulated genes after remission. **(E)** Venn diagram showing the overlap between fusion genes and downregulated genes after remission. **(F)** Volcano map comparing the gene expression profiles in the high-risk and non-high-risk groups. **(G)** The most highly upregulated pathways in the high-risk group. **(H)** The most downregulated pathways in the high-risk group.** (I) **Venn diagram showing the overlap between the fusion genes and upregulated genes in the high-risk group after remission. **(J)** Venn diagram showing the overlap between the fusion genes and downregulated genes in the high-risk group after remission. K. Fusion genes in RAS signaling pathway-mutated B-ALL

RAS signaling pathway gene mutations are controversial factors regarding patient prognosis. Thus far, we do not know the necessity of targeted therapy for ALL patients with RAS signaling pathway gene mutations, nor do we know the targets that should be focused on for ALL patients with RAS signaling pathway gene mutations. Therefore, we investigated the general characteristics of the RAS signaling pathway gene mutation subgroup. Thirteen patients carrying RAS signaling pathway genes were identified in the cohort, 4 of which presented high-risk characteristics, while 9 presented of nonhigh-risk characteristics. There were 193 upregulated genes and 108 downregulated genes in the RAS signaling pathway gene mutation subgroup (Fig. 6A). The pathways involved are illustrated in Fig. 6B-C. By comparing the overlap between these DEGs and fusion genes, we identified several shared genes (Fig. 6D and E). However, the overlaps were not significant.

For comparison between the RAS signaling pathway genes mutated high-risk and RAS pathway genes mutated non-high-risk, there were 193 upregulated genes and 108 downregulated genes in the RAS signaling pathway gene mutation subgroup (Fig. 6F). The pathways involved are illustrated in Fig. 6G-H. By comparing the overlap between these DEGs and fusion genes, we identified several shared genes (Fig. 6I and J). However, the overlaps were not significant. Interestingly, the fusion genes *MRPL13::ZNF740* (*n* = 4), *DHRSX* duplication (*n* = 4), *CYR5A::DIPK1C* (*n* = 4) and *CXorf21::MRPS16A* (*n* = 3) were dominant in RAS signaling pathway-mutated B-ALL **(**Fig. 6K).

**Fig. 7:**
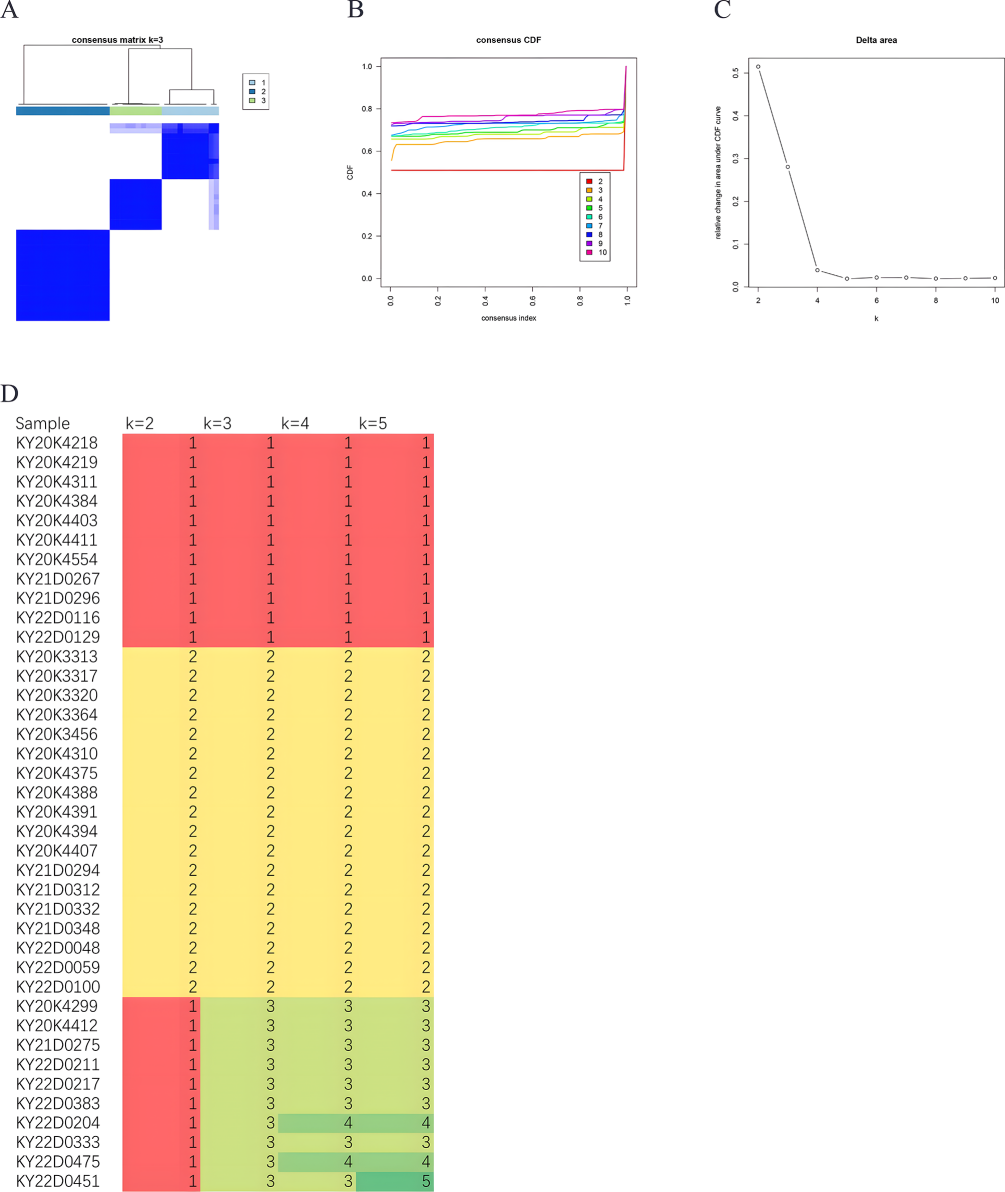
Molecular subtyping according to expression profiles in 45 pediatric B-ALL patients in the cohort. **A. **According to the molecular subtyping according to the expression profiles, the patients are divided into three major subgroups. **B-C. **When the subgroup number was increased from 3 to 10, the AUC of the consistency index between K = 3 and K = 10 was not significantly different. **D.** Genetic expression profiles were compared among these three subgroups. Different colors indicate different numbers, with red indicating 1, yellow indicationg 2, light-green indicating 3 and green indicating 4

**Fig. 8:**
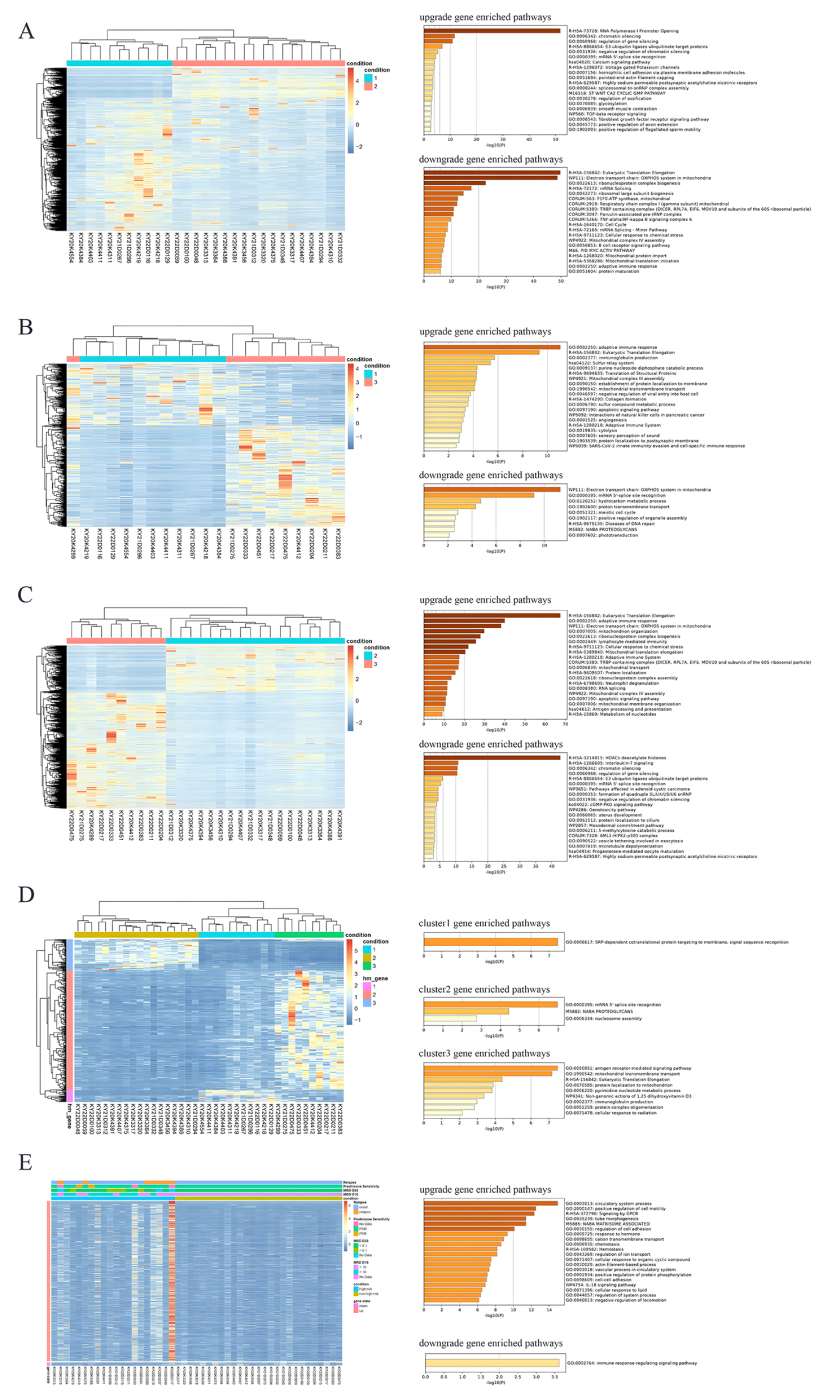
Expression profiles in molecular subgroups. **A-C**. Pairwise comparison of expression profiles between the two subgroups and the involved pathways. **D**. Comparison of expression profiles among the three subgroups and the involved pathways. **E.** Validation of the molecular subtyping model by comparing MRD and prednisone response between clusters and subgroups

For B-ALL without known molecular markers, molecular subtyping according to expression profiles would help identify new subgroups (Fig. 7). The AUCs of the consistency indices between K = 3 and K = 10 were not significantly different (Fig. 7A-C). Therefore, they were subtyped into three groups: Group 1, Group 2 and Group 3 (Fig. 7D). Differentially expressed genes were compared among these subgroups. A pairwise comparison of the three subgroups is illustrated in Tables 3 and Fig. 8. A comparison of Group 1 and Group 2, revealed and the DEGs and pathways involved in these genes affected RNA polymerase I promoter opening, eukaryotic translation elongation, and the electron transport chain of the OXPHOS system in mitochondria (Fig. 8A). A comparison of Group 1 and Group 3 revealed that the DEGs and involved pathways affected eukaryotic translation elongation and the electron transport chain of the OXPHOS system in mitochondria **(**Fig. 8B). The expression profiles of Group 1 and Group 3 were very similar, as shown by the transcriptome data. Group 3 should be regarded as deriving from Group 1. A comparison of Group 2 and Group 3 revealed that the DEGs and pathways involved affect eukaryotic translation elongation and that HDACs deacetylate histones (Fig. 8C). Gene Cluster 1, involving pathways of SRP-dependent cotranslational protein targeting membrane and signal sequence recognition, was upregulated in Group 1. Gene Cluster 2, involving pathways of mRNA 5’-splice site recognition pathways, NABA proteoglycans, and nucleosome assembly, was upregulated in Group 3. The expression of genes in Cluster 3, involving antigen receptor-mediated signaling pathway, mitochondrial transmembrane transport, eukaryotic translation elongation, and mitochondrial protein pathways, was upregulated in Group 2 (Fig. 8D).

**Table 3:** Comparison for gene expression across groups of different mRNA expression profiles

Paired groups	Number of up-regulated genes	Number of down-regulated genes
Group 1 vs. Group 2	4893(2303)	1292(1059)
Group 1 vs. Group 3	489(400)	781(323)
Group 2 vs. Group 3	2589(2331)	6255(2974)

Note: In parentheses are the number of annotated genes

To determine whether these pathways are related to the therapeutic response, we further validated the model by comparing MRD and prednisone responses between clusters and subgroups (Fig. 8E). Group 1 is more likely to be high-risk, while Group 3 is less likely to be high-risk. Although these two groups showed similar downregulated transcriptomes, the differential expression of gene Cluster 3 was likely the most important factor related to the different treatment responses. The upregulated pathways in the high-risk group were related to the circulatory system process, positive regulation of cell motility, signaling by GPCRs, tube morphogenesis, *NABA* microsome association, regulation of cell adhesion, and response to the hormones. The downregulated pathways in the high-risk group were related to the immune response-regulating signaling pathway. To eliminate the bias caused by mutations of P16 deletion, *KRAS* mutations, and *NRAS* mutations, which were frequent in the cohort, the distribution differences were compared between subgroups, and the results presented no significant difference (*p* > 0.05).

## Discussion

This is an exploratory study, with the main finding that RNA sequencing can reveal previously unreported fusion genes in some pediatric B-ALL patients whose chemotherapy response is inconsistent with the risk stratification corresponding to routine molecular biological features. Abnormal regulation of previously neglected signaling pathways can also be found. These new abnormalities may be related to pathogenesis and treatment response. Further comparison of groups of patients with different treatment responses may reveal new classifications of expression profiles. A novel approach for targeted treatment of B-ALL based on the transcriptome is discussed.

In a previous study, according to the transcriptional landscape, B-ALL patients were divided into subgroups with different fusion genes and known gene mutations, which included *MEF2D* fusions, *TCF3-PBX1, ETV6::RUNX1 or ETV6::RUNX1-like, DUX4* fusions, *ZNF384* fusions, *BCR::ABL1 or BCR::ABL1*-like, high hyperdiploidy, *KMT2A* fusions, *PAX5* and *CRLF2* fusions, *PAX5* (p. P80R) mutations, *IKZF1* (p. N159Y) mutations, *ZEB2 (p. H1038R)/IGH::CEBPE*, *TCF3/4::HLF*, and *NUTM1* fusions [3]. Gene mutations among signaling molecules, epigenetic factors, and transcription factor genes are enriched in different subgroups, which means that, except for subgroup identifiers, enriched mutations in different functional genes are attributed to the gene expression files together [23]. However, regarding a random individual patient, it does not always copy the genomic and epigenetic characteristics of certain subgroups, and there would be confounding variations [24–28]. It is still necessary to discover the underlying genomic defects and mysterious mechanisms in individuals for treatment response [29]. By using an intertomics-based approach, transcriptome-based subcluster identification can be achieved directly at the clinical level, which may be critical for predicting potential drug targets for individual subclusters [5]. Therefore, RNA-seq can be implemented in the diagnostic workflow of ALL and enhances the individual molecular diagnostic risk classification of ALL [4, 30].

In this study, we explored the gene expression pattern in a group of childhood B-ALL patients whose molecular biological characteristics and chemotherapy response did not completely match the above subgroup identifiers. RNA-seq-based subgroups were used stratify patients into low-risk, intermediate-risk, and high-risk groups according to survival [3]. *BCR::ABL1*-positive B-ALL is usually regarded as a “high-risk” reference [31–33], while *ETV6::RUNX1*-positive B-ALL is a “low-risk” reference [34]. Subgroups with *TCF3::PBX1, ETV6::RUNX1*-like, *ZNF384* fusions, *DUX4* fusions [34], or high hyperdiploidy are low-risk according to the *BCR::ABL1*-positive subgroup and *ETV6::RUNX1*-positive subgroup. *PAX5* and *CRLF2* fusions and hyperdiploidy (≤ 50 chromosomes) are classified into the intermediate-risk group due to an inferior 5-year overall survival compared with that of *ETV6::RUNX1*-positive/*ETV6::RUNX1*-like. *MEF2D* fusions and *KMT2A* fusions tend to be associated with high risk [3]. Usually, the low-risk group responds well to chemotherapy. It does not need highly intensified treatment to induce or maintain long-term remission [35, 36]. In contrast, the high-risk group is often insensitive to chemotherapy and needs intensified treatment with chemotherapy, immunotherapy, or small molecular targeted medicine [37]. We did not stratify B-ALL patients simply according to the above criteria. Post-chemotherapy treatment response evaluation through prednisone response and/or MRD corresponds well to the long-term prognosis. Therefore, in this study, we retrospectively evaluated the included patients in terms of prednisone response and MRD status, ignoring the molecular biological characteristics indentified by the enrollment test, to analyze the transcriptome characteristics related to treatment response-.

Gene expression profiling has revealed new cytogenetic subgroups that display certain specific gene expression patterns. In this study, DNA methylation played a very special role in this cohort. Moreover, the relationships between genes involved in homophilic cell adhesion via plasma membrane adhesion molecules, development growth, regulation of cell differentiation, B-cell proliferation, and leukemia progression in the cohort are not well understood and should be further evaluated for treatment designation. There is a potential role for DNA methylation-targeting therapy in this cohort. Furthermore, pathways related to exocytosis, cell chemotaxis, inflammatory response, and cell proliferation and survival were upregulated, which was probably attributed to leukemia stem cell and relapse clone retention in the high-risk group. The suppressed pathways related to the regulation of ion transmembrane transport, potassium ion transmembrane transport, negative regulation of cellular amide metabolic processes, and protein‒DNA complex assembly likely suppress protein metabolism and provide self-protection.

RAS signaling pathway mutational status of *NRAS*, *KRAS*, and *PTPN11* genes is associated with genetic/cytogenetic features in children with B-precursor acute lymphoblastic leukemia. Previous reports have shown that 70% of ALL patients with damaging germline *ETV6* variants exhibit hyperdiploid karyotypes with characteristic recurrent mutations in *NRAS*, *KRAS*, and *PTPN11* [38]. The RAS signaling pathway is related to the pathogenesis, prognosis, and relapse process of B-ALL. However, in the analysis of the subgroup expression profile of the cohort, we found that the distribution of RAS signaling pathway mutations among subgroups was not significantly different, which indicated that RAS signaling pathway mutations did not play key roles in controlling the variations in the expression profile. This finding does not support the idea that the mutation status of the RAS signaling pathway may be involved in therapy designation in the future.

Fusion gene detection of candidate pharmacogenes/pharmacotranscripts cannot revealed new genes or transcripts [21]. The transcriptomes of fusion genes may be more informative of disease development, cell proliferation, differentiation, and apoptosis. In this study, we identified new fusion genes without identifying their biological functions. Seven patients in the high-risk group carried *DHRSX* duplications. According to a previous report, using overexpression and knockdown analyses, Zhang et al. showed that *DHRSX* promoted starvation-induced autophagy in HeLa and U2OS cells [39]. The promotion of autophagy by *DHRSX* involves the downregulation of AKT/mTOR phosphorylation and the upregulation of beclin-1, which are highly related to the antiapoptotic function and chemotherapy resistance of leukemia cells [40–42]. This result provides new insight for further exploration of a new mechanism for drug resistance in B-ALL. Whether *DHRSX* duplication enhances the function of starvation-induced autophagy in B-ALL remains to be determined in future studies.

Although ALL patients are stratified according to genomic information, we cannot adjust the therapy without MRD evaluation at the end of induction therapy [2, 8, 9]. Treatment of childhood refractory and relapsed acute lymphoblastic leukemia (R/R ALL) patients with refractory and poor prognostic genes cannot be resolved by chemotherapy alone [43]. In recent years, small-molecule targeted drugs have been proven effective in clinical trials [44–48]. Small molecule targeted drugs regulate a variety of signaling pathways by targeting signaling molecules that play a regulatory role in cell proliferation, differentiation, and apoptosis [10–19]. For example, targeting *BCR::ABL1* and *BCR::ABL1*-like [29, 46, 49–51] significantly affects patient prognosis. Therefore, RNA-seq-based therapy adjustments may be more beneficial in the *ETV6::RUNX1*-positive subgroup. The “low-risk” *ETV6::RUNX1*-positive subgroup was not always low-risk, similar to the patients in this study. It has been reported that the intensity of chemotherapy [52] in the *ETV6::RUNX1*-positive subgroup should not decrease for those who have positive MRD postinduction chemotherapy. Discontinuation of L-asparaginase and poor response to prednisolone is associated with poor outcomes in *ETV6::RUNX1*-positive pediatric B-ALL patients, indicating drug insensitivity [53]. Autophagy inhibition [54], spleen tyrosine kinase (SYK) [55], and *IGF2BP1* [56] are potential future therapeutic targets for *ETV6::RUNX1*-driven B-cell precursor acute lymphoblastic leukemia due to their roles in *ETV6::RUNX1* cell survival and prognosis. Through RNA-seq, we can identify upregulated and activated pathways at diagnosis and design combination chemotherapeutics to sensitize resistant primary cells to conventional drugs. There were 4 *ETV6::RUNX1*-positive high-risk patients for whom further analysis of the mechanism underlying the poor response to therapy was needed. For those with drug-resistant ALL, further exploration of the gene expression patterns of leukemic cells and translation of gene-gene interactions would be meaningful for gene expression pattern-based drug combination therapy development.

## Conclusions

Although large cohort studies have systematically classified ALL, some patients often cannot use these classification models to explain their illness and treatment response. When their insensitivity to treatment cannot be explained by the existing classification system, we can analyze their expression profiles through RNA-seq by implementing a diagnostic system to identify activated markers related to leukemogenesis and drug resistance. These findings will guide the selection of targeted drugs and the design of treatment plans in the future. The implementation of an RNA-seq diagnosis system and a new concept for treatment deserve a prospective study.

### Electronic supplementary material

Below is the link to the electronic supplementary material.

Supplementary Material 1

## Data Availability

No datasets were generated or analysed during the current study.

## References

[1] Li XY, Li JQ, Luo XQ (2021). Reduced intensity of early intensification does not increase the risk of relapse in children with standard risk acute lymphoblastic leukemia - a multi-centric clinical study of GD-2008-ALL protocol. BMC Cancer.

[2] Borowitz MJ, Wood BL, Devidas M (2015). Prognostic significance of minimal residual disease in high risk B-ALL: a report from Children’s Oncology Group study AALL0232. Blood.

[3] Li JF, Dai YT, Lilljebjorn H (2018). Transcriptional landscape of B cell precursor acute lymphoblastic leukemia based on an international study of 1,223 cases. Proc Natl Acad Sci U S A.

[4] Brown LM, Lonsdale A, Zhu A (2020). The application of RNA sequencing for the diagnosis and genomic classification of pediatric acute lymphoblastic leukemia. Blood Adv.

[5] Mukherjee S, Kar A, Paul P (2022). Silico Integration of Transcriptome and Interactome predicts an ETP-ALL-Specific transcriptional footprint that decodes its Developmental Propensity. Front Cell Dev Biol.

[6] Dai YT, Zhang F, Fang H (2022). Transcriptome-wide subtyping of pediatric and adult T cell acute lymphoblastic leukemia in an international study of 707 cases. Proc Natl Acad Sci U S A.

[7] Wang Q, Cai WZ, Wang QR (2023). Integrative genomic and transcriptomic profiling reveals distinct molecular subsets in adult mixed phenotype acute leukemia. Am J Hematol.

[8] Roberts KG, Pei D, Campana D (2014). Outcomes of children with BCR-ABL1-like acute lymphoblastic leukemia treated with risk-directed therapy based on the levels of minimal residual disease. J Clin Oncol.

[9] Mullighan CG, Jeha S, Pei D (2015). Outcome of children with hypodiploid ALL treated with risk-directed therapy based on MRD levels. Blood.

[10] Kurosu T, Ohki M, Wu N (2009). Sorafenib induces apoptosis specifically in cells expressing BCR/ABL by inhibiting its kinase activity to activate the intrinsic mitochondrial pathway. Cancer Res.

[11] Pratz KW, Cho E, Levis MJ (2010). A pharmacodynamic study of sorafenib in patients with relapsed and refractory acute leukemias. Leukemia.

[12] Borthakur G, Kantarjian H, Ravandi F (2011). Phase I study of sorafenib in patients with refractory or relapsed acute leukemias. Haematologica.

[13] Wakim JJ, Tirado CA, Chen W et al. *t(8;22)/BCR-FGFR1 myeloproliferative disorder presenting as B-acute lymphoblastic leukemia: report of a case treated with sorafenib and review of the literature* Leuk Res, 2011. 35(9): pp. e151-3. 10.1016/j.leukres.2011.05.013 21628071

[14] Walz C, Erben P, Ritter M (2011). Response of ETV6-FLT3-positive myeloid/lymphoid neoplasm with eosinophilia to inhibitors of FMS-like tyrosine kinase 3. Blood.

[15] Usuki K, Tojo A, Maeda Y (2012). Efficacy and safety of nilotinib in Japanese patients with imatinib-resistant or -intolerant Ph + CML or relapsed/refractory Ph + ALL: a 36-month analysis of a phase I and II study. Int J Hematol.

[16] Ottosson-Wadlund A, Ceder R, Preta G (2013). Requirement of apoptotic protease-activating factor-1 for bortezomib-induced apoptosis but not for Fas-mediated apoptosis in human leukemic cells. Mol Pharmacol.

[17] Shimoni A, Volchek Y, Koren-Michowitz M (2015). Phase 1/2 study of nilotinib prophylaxis after allogeneic stem cell transplantation in patients with advanced chronic myeloid leukemia or Philadelphia chromosome-positive acute lymphoblastic leukemia. Cancer.

[18] Papadantonakis N, Advani AS (2016). Recent advances and novel treatment paradigms in acute lymphocytic leukemia. Ther Adv Hematol.

[19] Bataller A, Garrote M, Oliver-Caldes A et al. Early T-cell precursor lymphoblastic leukaemia: response to FLAG-IDA and high-dose cytarabine with sorafenib after initial refractoriness. Br J Haematol, 2018. 10.1111/bjh.15601 30334573

[20] Pavlovic S, Kotur N, Stankovic B et al. Pharmacogenomic and pharmacotranscriptomic profiling of Childhood Acute Lymphoblastic Leukemia: paving the way to Personalized Treatment. Genes (Basel), 2019. 10(3). 10.3390/genes10030191 PMC6471971 30832275

[21] Amos W, Driscoll E, Hoffman JI (2011). Candidate genes versus genome-wide associations: which are better for detecting genetic susceptibility to infectious disease?. Proc Biol Sci.

[22] Wilkerson MD, Hayes DN (2010). ConsensusClusterPlus: a class discovery tool with confidence assessments and item tracking. Bioinformatics.

[23] Janczar K, Janczar S, Pastorczak A (2015). Preserved global histone H4 acetylation linked to ETV6-RUNX1 fusion and PAX5 deletions is associated with favorable outcome in pediatric B-cell progenitor acute lymphoblastic leukemia. Leuk Res.

[24] Masuda T, Maeda S, Shimada S (2022). RUNX1 transactivates BCR-ABL1 expression in Philadelphia chromosome positive acute lymphoblastic leukemia. Cancer Sci.

[25] Mian AA, Zafar U, Ahmed SMA (2021). Oncogene-independent resistance in Philadelphia chromosome - positive (Ph(+)) acute lymphoblastic leukemia (ALL) is mediated by activation of AKT/mTOR pathway. Neoplasia.

[26] Gu J, Reynolds A, Fang L (2016). Coexistence of iAMP21 and ETV6-RUNX1 fusion in an adolescent with B cell acute lymphoblastic leukemia: literature review of six additional cases. Mol Cytogenet.

[27] Lee JW, Kim S, Jang PS (2021). Differing outcomes of patients with high Hyperdiploidy and ETV6-RUNX1 rearrangement in Korean Pediatric Precursor B Cell Acute Lymphoblastic Leukemia. Cancer Res Treat.

[28] Dun KA, Vanhaeften R, Batt TJ (2016). BCR-ABL1 gene rearrangement as a subclonal change in ETV6-RUNX1-positive B-cell acute lymphoblastic leukemia. Blood Adv.

[29] Kaczmarska A, Sliwa P, Zawitkowska J et al. Genomic analyses of Pediatric Acute Lymphoblastic Leukemia Ph + and Ph-Like-recent progress in treatment. Int J Mol Sci, 2021. 22(12). 10.3390/ijms22126411 PMC8232636 34203891

[30] Schieck M, Lentes J, Thomay K (2020). Implementation of RNA sequencing and array CGH in the diagnostic workflow of the AIEOP-BFM ALL 2017 trial on acute lymphoblastic leukemia. Ann Hematol.

[31] Zhang Z, Chen Z, Jiang M (2019). Heterogeneous BCR-ABL1 signal patterns identified by fluorescence in situ hybridization are associated with leukemic clonal evolution and poorer prognosis in BCR-ABL1 positive leukemia. BMC Cancer.

[32] Zhang L, Ramjit RT, Hill CE (2016). Clinical significance of quantitative monitoring and mutational analysis of BCR-ABL1 transcript in Philadelphia chromosome positive B lymphoblastic leukemia. Leuk Lymphoma.

[33] Soverini S, Albano F, Bassan R (2020). Next-generation sequencing for BCR-ABL1 kinase domain mutations in adult patients with Philadelphia chromosome-positive acute lymphoblastic leukemia: a position paper. Cancer Med.

[34] Burmeister T, Gokbuget N, Schwartz S (2010). Clinical features and prognostic implications of TCF3-PBX1 and ETV6-RUNX1 in adult acute lymphoblastic leukemia. Haematologica.

[35] Hoffmann J, Krumbholz M, Gutierrez HP (2019). High sensitivity and clonal stability of the genomic fusion as single marker for response monitoring in ETV6-RUNX1-positive acute lymphoblastic leukemia. Pediatr Blood Cancer.

[36] Bhojwani D, Pei D, Sandlund JT (2012). ETV6-RUNX1-positive childhood acute lymphoblastic leukemia: improved outcome with contemporary therapy. Leukemia.

[37] King AC, Pappacena JJ, Tallman MS (2019). Blinatumomab administered concurrently with oral tyrosine kinase inhibitor therapy is a well-tolerated consolidation strategy and eradicates measurable residual disease in adults with Philadelphia chromosome positive acute lymphoblastic leukemia. Leuk Res.

[38] Liang DC, Chen SH, Liu HC et al. Mutational status of NRAS, KRAS, and PTPN11 genes is associated with genetic/cytogenetic features in children with B-precursor acute lymphoblastic leukemia. Pediatr Blood Cancer, 2018. 65(2). 10.1002/pbc.26786 28853218

[39] Zhang G, Luo Y, Li G (2014). DHRSX, a novel non-classical secretory protein associated with starvation induced autophagy. Int J Med Sci.

[40] Wu X, Feng X, Zhao X (2016). Role of beclin-1-Mediated autophagy in the Survival of Pediatric Leukemia cells. Cell Physiol Biochem.

[41] Neri LM, Cani A, Martelli AM (2014). Targeting the PI3K/Akt/mTOR signaling pathway in B-precursor acute lymphoblastic leukemia and its therapeutic potential. Leukemia.

[42] Zhang J, Liu X, Yin C (2022). hnRNPK/Beclin1 signaling regulates autophagy to promote imatinib resistance in Philadelphia chromosome-positive acute lymphoblastic leukemia cells. Exp Hematol.

[43] Vrooman LM, Blonquist TM, Harris MH (2018). Refining risk classification in childhood B acute lymphoblastic leukemia: results of DFCI ALL Consortium Protocol 05 – 001. Blood Adv.

[44] Short NJ, Kantarjian H, Jabbour E (2021). Optimizing the treatment of acute lymphoblastic leukemia in younger and older adults: new drugs and evolving paradigms. Leukemia.

[45] Hughes TP, Laneuville P, Rousselot P (2019). Incidence, outcomes, and risk factors of pleural effusion in patients receiving dasatinib therapy for Philadelphia chromosome-positive leukemia. Haematologica.

[46] Braun TP, Eide CA, Druker BJ (2020). Response and resistance to BCR-ABL1-Targeted therapies. Cancer Cell.

[47] Bahjat M, de Wilde G, van Dam T (2019). The NEDD8-activating enzyme inhibitor MLN4924 induces DNA damage in Ph + leukemia and sensitizes for ABL kinase inhibitors. Cell Cycle.

[48] Korfi K, Smith M, Swan J (2016). BIM mediates synergistic killing of B-cell acute lymphoblastic leukemia cells by BCL-2 and MEK inhibitors. Cell Death Dis.

[49] Piccaluga PP, Paolini S, Martinelli G (2007). Tyrosine kinase inhibitors for the treatment of Philadelphia chromosome-positive adult acute lymphoblastic leukemia. Cancer.

[50] Cario G, Leoni V, Conter V (2020). Relapses and treatment-related events contributed equally to poor prognosis in children with ABL-class fusion positive B-cell acute lymphoblastic leukemia treated according to AIEOP-BFM protocols. Haematologica.

[51] Montecchini O, Braidotti S, Franca R (2021). A novel ELISA-Based peptide Biosensor Assay for Screening ABL1 activity in vitro: a challenge for Precision Therapy in BCR-ABL1 and BCR-ABL1 like Leukemias. Front Pharmacol.

[52] Takahashi Y, Ishida H, Imamura T (2022). JACLS ALL-02 SR protocol reduced-intensity chemotherapy produces excellent outcomes in patients with low-risk childhood acute lymphoblastic leukemia. Int J Hematol.

[53] Usami I, Imamura T, Takahashi Y (2019). Discontinuation of L-asparaginase and poor response to prednisolone are associated with poor outcome of ETV6-RUNX1-positive pediatric B-cell precursor acute lymphoblastic leukemia. Int J Hematol.

[54] Polak R, Bierings MB, van der Leije CS (2019). Autophagy inhibition as a potential future targeted therapy for ETV6-RUNX1-driven B-cell precursor acute lymphoblastic leukemia. Haematologica.

[55] Serafin V, Porcu E, Cortese G et al. SYK Targeting represents a potential therapeutic option for relapsed resistant Pediatric ETV6-RUNX1 B-Acute lymphoblastic leukemia patients. Int J Mol Sci, 2019. 20(24). 10.3390/ijms20246175 PMC6940898 31817853

[56] Sharma G, Boby E, Nidhi T (2021). Diagnostic utility of IGF2BP1 and its targets as potential biomarkers in ETV6-RUNX1 positive B-Cell Acute Lymphoblastic Leukemia. Front Oncol.

