# Supplementary material for: The potential role of RNA sequencing in diagnosing unexplained insensitivity to conventional chemotherapy in pediatric patients with B-cell acute lymphoblastic leukemia
Source: BMC Med Genomics. 2024 May 29;17:149. doi: 10.1186/s12920-024-01892-w (PMC11137891; doi:10.1186/s12920-024-01892-w)
Supplement: Supplementary file 1 — Supplementary Material 1 [file 12920_2024_1892_MOESM1_ESM.docx]

Supplementary information

SI 1. The risk classification of SCCLG-2016-ALL protocol

Evaluation of treatment response and efficacy:

① Prednisone response test: Patients with absolute peripheral blood neoplasm lymphocyte count < 1×10^9^ /L on day 8 of prednisone induction were classified as prednisone good response (PGR), and patients with ≥1 ×10^9^ /L were classified as prednisone poor response (PPR);

② Bone marrow smear results on days 15 and 33: marrow neoplasm cells < 5% were defined as M1, 5%-25% as M2, and > 25% as M3.

③ Rapid early responses were defined as bone marrow MRD < 0.1% on day 15 of induction chemotherapy; Slow early responses were defined as bone marrow MRD≥0.1% on day 15 of induction.

④ Central nervous system leukemia (CNSL)is diagnosed CNSL on anyone of the following conditions: ⑴ in cerebrospinal fluid ,WBC > 5/μ L with the majority of white blood cells, at the same time red blood cell/white blood cell ratio ≤ 100:1; Or cerebrospinal fluid WBC > 5/μ L, in which the percentage of white blood cell count was higher than the percentage of neoplasm cells in the peripheral blood; Or cerebrospinal fluid WBC > 5/μ L and flow cytometry positive; ⑵ Cranial nerve palsy; (3) CT/MRI showed cerebral or meningeal lesions.

⑤ relapse: includes bone marrow relapse, central nervous system relapse, testicular relapse and combined relapse. Bone marrow relapse is when the ratio of neoplasm cells in bone marrow > 25% after complete remission of ALL. Central nervous system relapse refers to neoplasm cells detected by centrifugal smear of cerebrospinal fluid or neoplasm infiltration in central nervous system, which cannot be explained by other reasons, or CT/MRI showing brain or meningeal lesions. Testicular relapse refers to ultrasound or biopsy confirming infiltration of unilateral or bilateral testicular leukemia cells. Combined relapse, i.e., extramedullary relapse (central and/or testicular relapse) with leukemia cells in bone marrow >5%.

⑥ Events include: persistent remission failure, relapse, occurrence of a second tumor, death, etc.

⑦ MRD monitoring: In this study, MRD was monitored during induction chemotherapy, and bone marrow MRD was evaluated on the 15th and 33rd day of induction chemotherapy, respectively. Positive D15 MRD was defined as MRD≥0.1%, and positive D33 MRD was defined as MRD≥0.01%. BC Navios flow cytometry was used to detect antibodies including CD45, CD34, CD117, CD38, CD123, TdT, CD33, CD13, CD15, CD5, CD7, CD19, CD10. The B-ALL combination also included cCD79a, cIgM, CD22, CD20, CD58, HLA-DR and CXCR4, while the T-ALL combination included cCD3, CD2, CD4, CD8, mCD3, CD57, CD1a, CD56 and CD16. CD45/ SSC gate method was used to distinguish normal and abnormal cell populations. Normal cell population was used as internal control to determine the expression of abnormal cell antigen. Immunophenotypic analysis of abnormal cell population was performed and the diagnosis was finally concluded.

SI 2. The details of sample preparation, RNA extraction, library preparation, and sequencing parameters

1. Sample preparation

(1) Collection of specimens

1) After admission, the patient signed the Informed Consent for Clinical Information and Sample Collection.

2) Samples from each patient should be collected at the time points of onset and remission, and relapse samples should be collected in case of relapse. Initial and recurrent specimens were collected after admission and before chemotherapy began, while remission specimens were collected when the patient's blood image recovered before consolidation chemotherapy.

3) Three EDTA tubes of 2ml bone marrow were routinely taken from each patient. If bone marrow volume was not enough, peripheral blood was collected according to the count of peripheral blood white blood cells: if white blood cells >5×10^9^/L, 3 tubes of 3mL bone marrow were extracted; If WBC <5×10^9^/L, 4 tubes of 3ml bone marrow were extracted. The bone marrow was temporarily stored at 4℃.

(2) Specimen handling

1) Sterilize the ultra-clean table and experimental items, rewarm the 4℃ stored lymphocyte separation solution in 37℃ water baths in advance, and rewarm the programmed cooling box.

2) Centrifuge sample tubes at 900rpm for 10min, carefully extract the plasma liquid at the top.

3) Add the same amount of sterile phosphoric acid buffer (PBC) to the remaining cell suspension, gently blow and mix.

4) Separation of cell components by density gradient centrifugation:

According to the sample size, the corresponding volume of lymphocyte separation solution was first added to the centrifugal tube, and the blood sample was carefully sucked with a Pasteurian pipette and added to the liquid surface of the separation solution, without oscillating or shaking, and the liquid surface should be maintained to form a clear dividing line. If using other brands of lymphocyte separations, read the instructions carefully to determine the ratio of lymphocyte separations to blood samples. Case A: When the blood sample size per tube was less than 3mL after dilution, the amount of lymphocyte separation solution was 3ml, 400-650g, centrifuged for 20-30min, slowly rising and falling. Case B: When the blood sample size was 3-10ml per tube after dilution, the same amount of separation solution was added to the blood sample, 450-650g, centrifuged for 20-30min, and the maximum centrifugal force was no more than 1200g, slowly rising and falling. Case C: When the blood sample size of each tube was 10-20ml after dilution, the separation solution of the same amount as the blood sample was added and centrifuged 500-1100g for 20-30min. The maximum centrifugal force was no more than 1200g, and the increase and decrease were slow. After centrifugation, the centrifugal tube is divided into four layers from top to bottom. The first layer is plasma layer, the second layer is annular milky lymphocyte layer, the third layer is transparent separation fluid layer, and the fourth layer is erythrocyte layer. The white membrane layer was carefully extracted by Pasteur tube and added into another 15ml centrifuge tube to obtain mononuclear cells. Appropriate amount of PBS was added, washed for 3 times, centrifuged at 1800rpm, and supernatant was discarded. After the third suspension with PBS, the cells were fully mixed for cell counting, and a tube of 1×10^7^ cells was separated and centrifuged separately. RNAiso Plus was added into the isolated tube of 1×10^7^ cells, which was fully mixed and transferred to the cryopreservation tube for LABELING RNA (for use in RNA-related experiments such as transcriptomic sequencing). Other cells were added with cryopreservation solution (10% DMSO+90% FBS) to make the cell density of 1×10^7^ cells/mL, with 5×10^6^ cells per tube, labeled PBMC (peripheral blood mononuclear cells). The transparent layer adjacent to the red blood cell layer was carefully sucked out with a pipette gun and added into another 15ml centrifuge tube to obtain neutrophils. Appropriate amount of PBS was added, washed for 3 times, centrifuged at 1800rpm, and the supernatant was discarded. After resuspended with PBS for the third time, cell count was fully mixed, and after centrifugation, the cell density was 1×10^7^ /mL, and frozen storage solution was added for resuspended. PMN (neutrophils) were labeled. 400ul of red blood cells were extracted with pipette gun, and the same amount of cryopreservation solution was added and mixed.

5) After programmed freezing, the cells are stored in a -80℃ dedicated sample box. The specimens should be marked with patients’ numbers, names, specimen types and collection date. The cell count in each PBMC, PMN and RNA tube and the identity of each tubes and the patients’ numbers were labeled outside the tubes.

(3) Storage of specimens

Specimens are stored in the biobank at -80℃ for a long time. The patients’ numbers, names, specimen types and tube numbers are carefully checked before shipment. The counting process is done quickly on ice or drikold. When the inventory is completed, the specimen bank staffs do the coding and storage.

2. RNA extraction

The reagents: Qubit RNA HS Assay Kit (Q32855, Life); Qubit dsDNA HS Assay Kit (Q32851, Life); NEBNext Ultra II Directional RNA Library Prep Kit for Illumina (#E7760, NEB); NEBNext rRNA Depletion Kit (#E6310, NEB); NovaSeq 5000/6000 S2 Reagent Kit (20012860, Illumina).

RNA was extracted using QIAGEN RNeasy Mini Kit according to the instructions.

3. Library construction

RRNA Depletion Kit was used to eliminate rRNA. Then use NEBNext Ultra Directional RNA Library Prep Kit II Directional RNA Library Prep Kit II Directional RNA Library Prep Kit ; Synthesis of cDNA second strand: The second strand synthesizes and deletes the original mRNA, producing double-stranded cDNA (DS cDNA); Purified double stranded cDNA: then the end of the double-stranded cDNA is repaired ,The repaired cDNA was then purified. Connect the sample connector, The DNA library attached to the connector is then purified.

4. Sequencing parameters

Fastq data was obtained by sequencing using NovaSeq 5000/6000 S2 Reagent Kit.
